# Effects of combined GnRHa and letrozole on serum steroid profiles in girls with advanced bone age

**DOI:** 10.3389/fendo.2026.1984633

**Published:** 2026-10-15

**Authors:** Xuan Zhang, Yanan Zhang, Yutong Xing, Wenqing Yang, Yueqiu Qin, Xuehan Gao, Xujing Kong, Susu Cai, Guocui Xue, Xiaomin Fan, Mingzhao Chen, Yongqi Liu, Baorong Chen, Xue Yan, Huifeng Zhang

**Affiliations:** 1 Department of Pediatrics, The Second Hospital of Hebei Medical University, Shijiazhuang, China; 2 Kingmed Diagnostics (Beijing) Co., Ltd, Beijing, China; 3 College of Chemistry, Beijing University of Chemical Technology, Beijing, China

**Keywords:** advanced bone age, girls, gonadotropin-releasing hormone agonist, letrozole, steroid hormones

## Abstract

**Objective:**

To evaluate the impact of combined GnRHa and letrozole on serum steroid profiles (classical, 11‐oxygenated, and backdoor androgen pathways) in girls with advanced bone age, and to monitor adverse events.

**Methods:**

28 girls with advanced bone age were enrolled. Of these, 15 received add‐on letrozole after GnRHa monotherapy (Group B); the remaining 13 started combination therapy from baseline (Group A). Blood was collected at baseline, 1, and 6 months. Steroids were measured by LC‐MS/MS.

**Results:**

At baseline, Group A had higher classical androgens, progestogens, estrogens, INB, SHBG, and FAI than Group B (all *P* < 0.05), while 11‐oxygenated androgens were similar. In Group A, classical androgens, P, 17‐OHP, 5α‐DHP, 17OH‐DHP, 3α‐diol, E1, E2, and FAI decreased markedly (all overall *P* < 0.01) and remained lower at 6 months; 11‐KT showed a modest reduction at 1 month (*P* = 0.02), but other 11‐oxygenated androgens were unchanged. In Group B, add‐on letrozole reduced E1, E2, AMH, and INB (all overall *P* < 0.05), while most androgens and progestogens were stable. No hyperandrogenism or adverse events were observed.

**Conclusions:**

Over 6 months, combined GnRHa and letrozole was not associated with hyperandrogenism in girls with advanced bone age. In Group A, gonadal androgens and estrogens were markedly suppressed, while adrenal androgens remained stable. In Group B, add‐on letrozole had limited effects on most androgens. Estrogen suppression was observed in both groups. These findings appear to support short‐term safety, although longer‐term data are needed.

## 1 Introduction

Rapidly progressive puberty (RPP) is characterized by timely pubertal onset but accelerated progression, leading to rapid bone age advancement and compromised predicted adult height (PAH) (1, 2). Although GnRHa is primarily indicated for central precocious puberty, the Chinese consensus on CPP (2022) recommends that girls with RPP may also be considered for GnRHa therapy when bone age advancement is rapid and height prognosis is severely compromised (2). However, clinical evidence suggests that GnRHa monotherapy may not adequately arrest bone age advancement in some patients, implying that estrogens may derive from sources beyond the gonads. Apart from direct ovarian secretion, peripheral conversion of adrenal‐derived androgen precursors by aromatase (CYP19A1) in peripheral tissues represents an additional source of estrogen. Aromatase inhibitors (AIs) block the conversion of C19 androgens to C18 estrogens, thereby slowing growth plate fusion—a mechanism that provides a rationale for their potential to improve adult height (3–5).

AIs have evolved into third‐generation agents, including non‐steroidal (e.g., letrozole and anastrozole) and steroidal compounds (e.g., exemestane). Among these, letrozole and anastrozole are the most commonly used for short stature in children (6). However, the lack of long‐term efficacy data remains a major limitation, and approximately one‐fifth of clinicians discontinue treatment due to adverse effects (7). Letrozole has been used predominantly in boys with conditions such as idiopathic short stature, constitutional delay of growth, and familial male‐limited precocious puberty. Its use in girls has been scarce, with few reports outside McCune‐Albright syndrome (7–9).

Theoretically, combining GnRHa with an AI may achieve dual suppression of estrogen signaling and more effectively slow bone age advancement. However, the use of letrozole in girls remains debated, with the core concern being androgen accumulation and hyperandrogenism (10). A 2016 study from Athens first investigated this approach in girls and found that GnRHa combined with anastrozole improved predicted adult height without abnormalities in testosterone, bone density, or spinal radiographs (11). A second-phase study further showed that continuing anastrozole monotherapy until a bone age of approximately 14 years optimized final height outcomes (12). These findings support the feasibility of this combination, though comprehensive steroid profiling was not performed in those studies.

Nevertheless, data remain limited regarding the steroidomic safety profile of combined GnRHa and letrozole—particularly its impact on dynamic changes in androgen metabolism—in girls, and high‐level evidence supporting its endocrine‐metabolic safety is still lacking. The present study therefore employed a prospective, self‐controlled design using liquid chromatography–tandem mass spectrometry (LC‐MS/MS) to comprehensively evaluate changes in serum steroid profiles—covering classical, 11‐oxygenated, and backdoor androgen pathways—in girls with RPP during combined GnRHa and letrozole therapy, and to assess treatment‐related safety parameters.

## 2 Materials and methods

### 2.1 Study design and participants

This study was approved by the Ethics Committee of the Second Hospital of Hebei Medical University (approval number: 2025‐R943). Written informed consent was obtained from the guardians of all participants.

Participants were consecutively enrolled between July 2025 and April 2026 from the outpatient clinic of the Department of Pediatric Endocrinology, Genetics and Metabolism at the Second Hospital of Hebei Medical University. Rapidly progressive puberty (RPP) was defined as accelerated sexual maturation (progression from one Tanner stage to the next within 3–6 months) and accelerated bone age maturation (bone age exceeding chronological age by more than 1 year), according to previously published criteria (1, 13). Exclusion criteria included conditions with precocious puberty that were not indications for GnRHa therapy, severe organ dysfunction, known drug allergy, androgen‐sensitive disorders, or concurrent participation in other interventional studies.

A total of 28 girls were enrolled: 13 initiated combination therapy with GnRHa and letrozole concurrently at baseline (Group A), and 15 received add‐on letrozole after established GnRHa monotherapy (Group B). These two groups were not prespecified study arms. Group A comprised patients who started GnRHa and letrozole together from the outset; Group B comprised patients who were already receiving GnRHa monotherapy and were subsequently offered add‐on letrozole. Assignment to either group was not based on disease severity, treatment response, or systematic allocation criteria, but reflected the timing of letrozole introduction relative to GnRHa initiation in each case.

### 2.2 Clinical assessment and follow‐up

All participants underwent systematic physical examinations and clinical data collection by professional physicians. Age, height, and weight were recorded, and body mass index was calculated. Pubertal development was assessed using Tanner staging. Hirsutism was evaluated using the modified Ferriman‐Gallwey score, with a score of ≥6 defined as hirsutism, and acne severity was documented using standard grading.

Peripheral venous blood was collected in the morning under fasting conditions. Serum samples were used to measure reproductive hormones, including anti‐Müllerian hormone, inhibin B, and sex hormone‐binding globulin, and to quantify steroid hormones by LC‐MS/MS (as described in section 2.3). The free androgen index was calculated as [total testosterone (nmol/L)/SHBG (nmol/L)] × 100.

All participants received a 6‐month regimen: GnRHa (triptorelin or leuprorelin) 80–100 μg/kg intramuscularly every 4 weeks, plus oral letrozole 1.25 mg once daily. Follow‐up blood sampling was scheduled at 1 and 6 months. Height, weight, and adverse events were documented at each visit, and left hand‐wrist radiographs were repeated at 6 months for bone age assessment.

### 2.3 Outcome measures and laboratory assays

Serum steroid hormones were measured by liquid chromatography–tandem mass spectrometry (LC‐MS/MS) using a laboratory‐developed test established in accordance with the CLSI C62 guideline (second edition) (14, 15). Separation was achieved on a Waters BEH C8+ column (2.1 mm × 100 mm, 1.7 μm) maintained at 50 °C, with gradient elution using 0.5 mM ammonium fluoride in water and methanol as mobile phases (16). The instrumentation consisted of a Waters UPLC‐MS/MS system (ACQUITY I‐Class/Xevo TQ‐S; Waters Corporation, USA). Mass spectrometry was performed with optimized parameters (capillary voltage 2.80 kV, ion source temperature 150 °C, desolvation temperature 500 °C). Method performance characteristics (precision, recovery, linear range, limit of detection, and limit of quantification) for each analyte, together with the elution gradient and mass spectrometric acquisition parameters, are provided in **Supplementary Tables 3–5**. Information on chemical reagents, standards, and internal standards is provided in **Supplementary Table 6**. The steroid panel measured covered the classical, 11‐oxygenated, and backdoor androgen pathways, along with progestogens, estrogens, and adrenal corticosteroids. For values below the lower limit of quantification, LOQ/2 was used for statistical analysis.

The laboratory (Beijing KingMed Diagnostics) is accredited by the China National Accreditation Service for Conformity Assessment under ISO 15189 (certificate number: MT0613). At least two concentration levels of internal quality controls were included in each batch, and clinical sample testing was performed only after quality control specifications were met. The laboratory participates annually in the external quality assessment program of the National Center for Clinical Laboratories; during the study period, all test items met the assessment criteria. In addition, selected steroids (progesterone, testosterone, estradiol, 17‐hydroxyprogesterone, aldosterone, cortisol, and estriol) participated in the IFCC‐RfB‐RELA inter‐laboratory comparison program, with all quality control results meeting the required standards.

Serum AMH, INB, and SHBG were measured by electrochemiluminescence immunoassay using a Roche E170 analyzer (Roche Diagnostics, Germany) at the Department of Clinical Laboratory of the Second Hospital of Hebei Medical University.

### 2.4 Statistical analysis

Data were first tested for normality using the Shapiro–Wilk method. Normally distributed data are presented as mean ± SD; non‐normally distributed data as median (25th, 75th percentile). For comparisons between two groups, the independent samples t‐test or Mann‐Whitney U test was used as appropriate. For variables with three repeated measurements, repeated‐measures ANOVA was used for overall comparisons when data were normally distributed at all time points, with Greenhouse–Geisser correction applied when Mauchly’s test was significant (*P* < 0.05), followed by paired t‐tests with Bonferroni correction. For variables with non‐normal distribution at any time point, the Friedman test was used, followed by Wilcoxon signed‐rank tests with Bonferroni correction. All reported pairwise *P* values are Bonferroni‐corrected, and *P* < 0.05 was considered statistically significant. All analyses were performed using SPSS version 26.0.

## 3 Results

### 3.1 Baseline characteristics

**Figure 1** depicts the complete steroid hormone biosynthetic network in females. It covers the classical androgen pathway (DHEA-S, DHEA, AD, TT), the 11-oxygenated androgen pathway (11-OHAD, 11-OHT, 11-KAD, 11-KT), and the backdoor pathway (5α-DHP, 17OH-DHP, 17OH-Allo, androsterone, androstanediol). Progestogens and estrogens are also included. Red arrows highlight the inhibitory sites of the combination therapy. GnRHa suppresses pituitary gonadotropin secretion. Letrozole blocks aromatase-mediated conversion of androgens to estrogens. This illustrates the dual regulatory effects of this regimen across the steroidogenic cascade.

**Figure 1:**
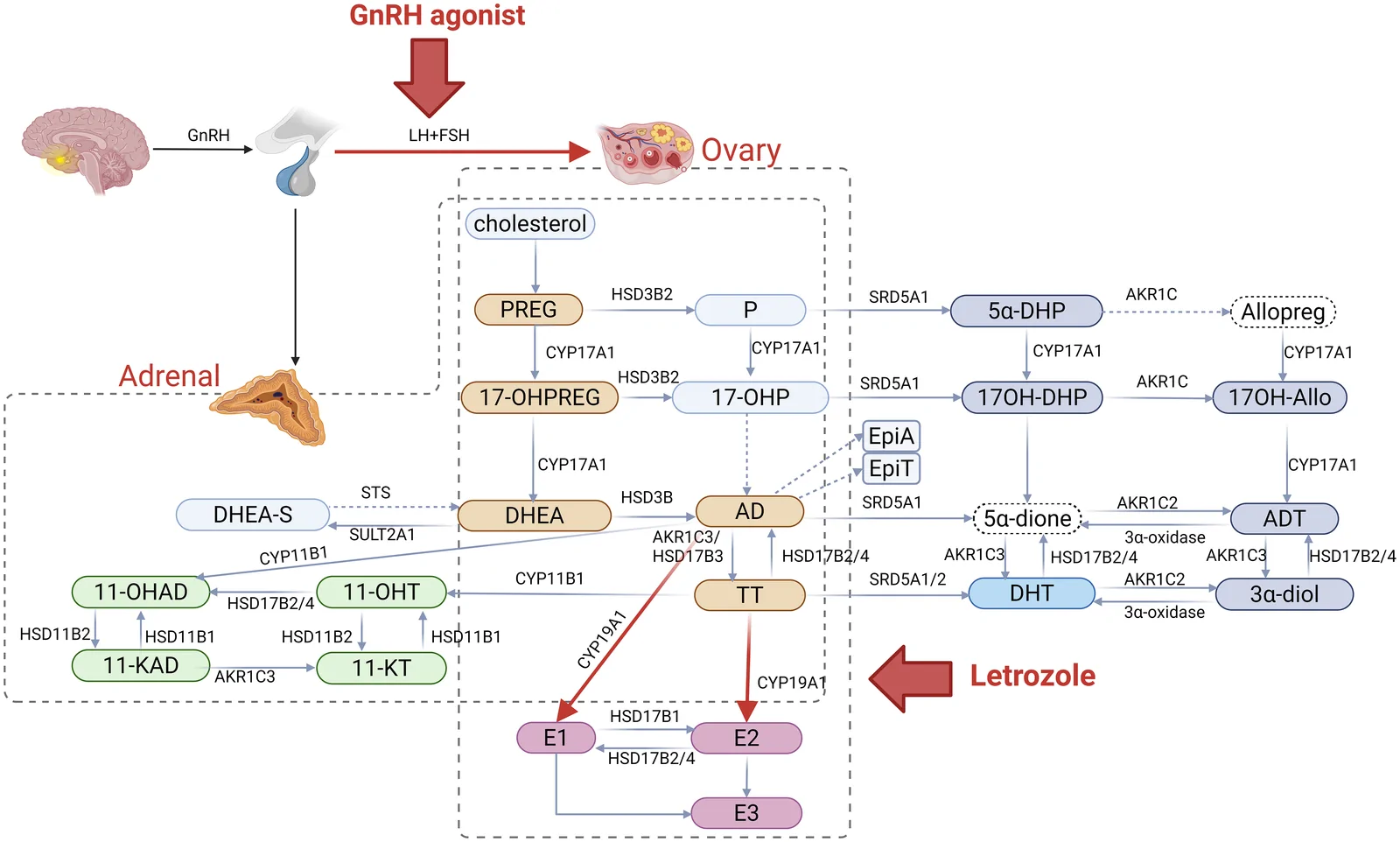
Schematic representation of the androgen, progestogen, and estrogen biosynthetic pathways in females, with annotated targets of combined GnRHa and letrozole therapy. Schematic of the steroidogenic pathways relevant to the present study. Yellow‐labeled hormones indicate the classical androgen pathway (DHEA‐S, DHEA, AD, TT); green‐labeled hormones indicate the 11‐oxygenated androgen pathway (11‐OHAD, 11‐OHT, 11‐KAD, 11‐KT); and gray‐labeled hormones indicate the backdoor pathway (5α‐DHP, 17OH‐DHP, 17OH‐Allo, 5α‐dione, ADT, 3α‐diol). Progestogens and estrogens are also depicted. Adrenal‐ and ovarian‐derived hormones partially overlap and are demarcated by dashed boxes, respectively. Red arrows indicate the sites of action of GnRHa (suppression of pituitary gonadotropin secretion) and letrozole (inhibition of aromatase), illustrating the dual regulatory effects of this combination regimen on the steroidogenic cascade. Of note, Allopreg and 5α‐dione, which are shown in the pathway diagram, were not measured in the present study. PREG, pregnenolone; P, progesterone; 17-OHPREG, 17-hydroxypregnenolone; 17-OHP, 17-hydroxyprogesterone; DHEA, dehydroepiandrosterone; DHEA-S, dehydroepiandrosterone sulfate; AD, androstenedione; TT, total testosterone; DHT, dihydrotestosterone; 11-OHAD, 11β-hydroxyandrostenedione; 11-KAD, 11-ketoandrostenedione; 11-KT, 11-ketotestosterone; 11-OHT, 11β-hydroxytestosterone; EpiA, epiandrosterone; EpiT, epitestosterone; 5α-DHP, 5α-dihydroprogesterone; Allopreg, allopregnanolone; 17OH-DHP, 17OH-dihydroprogesterone; 17OH-Allo, 17OH-allopregnanolone; 5α-dione, 5α-androstanedione; ADT, androsterone; 3α-diol, androstanediol; E1, estrone; E2, estradiol; E3, estriol. Created in BioRender. activity, P. (2026) https://BioRender.com/11cj7ti.

A total of 28 girls with advanced bone age were enrolled, comprising 13 in the Group A (de‐novo combination therapy) and 15 in the Group B (add‐on letrozole after GnRHa monotherapy). Baseline demographic and clinical characteristics are summarized in **Table 1**. Baseline demographic and clinical characteristics were comparable between the two groups, including age, height, and body weight (all *P* > 0.05). With respect to reproductive hormones, serum levels of INB, SHBG, and FAI were significantly higher in the Group A (*P* = 0.04, 0.02, and 0.03, respectively), whereas AMH did not differ significantly (*P* = 0.66). Among progestogens, the Group A exhibited markedly higher concentrations of P, 17‐OHP, 5α‐DHP, and 17OH‐DHP (all *P* < 0.05). In the androgen axis, all measured classical pathway androgens—including AD, TT, ADT, DHT, and EpiT—were significantly elevated in the Group A (all *P* < 0.05), whereas none of the 11‐oxygenated androgens showed significant intergroup differences (all *P* > 0.05). For estrogens, E1 and E2 were significantly higher in the Group A (both *P* < 0.01), while E3 was comparable between the two groups (*P* = 0.30). Overall, the Group A displayed a more active steroidogenic profile at baseline, particularly involving the classical androgen and estrogen pathways

**Table 1:** Baseline demographic and clinical characteristics of the girls with advanced bone age.

Variable	Group A (n=13)	Group B (n=15)	*P* value
Clinical characteristics			
Age (years)	11.58 ± 1.33^a^	11.43 ± 1.11^a^	0.74
Bone age (years)	12.53 ± 0.52^a^	12.42 ± 0.93^a^	0.71
Height (cm)	146.47 ± 7.82^a^	149.76 ± 4.54^a^	0.41
Body weight (kg)	43.18 ± 9.14^a^	42.57 ± 4.97^a^	0.83
Reproductive hormones			
AMH (ng/mL)	3.61 ± 1.62^a^	4.00 ± 2.85^a^	0.66
INB (pg/mL)	116.08 (65.68, 222.98)^b^	62.64 (50.50, 114.11)^b^	0.04
SHBG (nmol/L)	51.70 ± 18.71^a^	28.82 (21.43, 40.67)^b^	0.02
FAI	1.86 ± 1.02^a^	1.54 (0.94, 2.49)^b^	0.03
Progestogens			
PREG (nmol/L; to ng/dL: ×31.65)	0.68 ± 0.45^a^	0.36 (0.24, 0.51)^b^	0.15
P (nmol/L; to ng/dL: ×31.45)	0.54 (0.13, 3.02)^b^	0.10 (0.03, 0.10)^b^	<0.01
17-OHPREG (nmol/L; to ng/dL: ×33.25)	2.20 (1.44, 4.87)^b^	1.41 (0.87, 3.19)^b^	0.25
17-OHP (nmol/L; to ng/dL: ×33.05)	1.79 ± 1.24^a^	0.45 (0.39, 0.61)^b^	<0.01
5α-DHP (nmol/L; to ng/dL: ×31.65)	0.25 (0.21, 0.45)^b^	0.08 (0.06, 0.17)^b^	<0.01
17OH-DHP (nmol/L; to ng/dL: ×31.25)	0.00 (0.00, 0.03)^b^	0.00 (0.00, 0.00)^b^	0.02
17OH-Allo (nmol/L; to ng/dL: ×33.43)	0.12 ± 0.10^a^	0.06 (0.00, 0.12)^b^	0.23
Androgens			
DHEA (nmol/L; to ng/dL: ×28.84)	6.10 ± 3.54^a^	4.09 (3.50, 7.66)^b^	0.56
DHEA-S (μmol/L; to μg/dL: ×36.85)	1.99 (0.68, 2.51)^b^	1.83 (1.53, 2.42)^b^	0.73
AD (nmol/L; to ng/dL: ×28.64)	2.81 ± 1.16^a^	1.14 (0.87, 1.31)^b^	<0.01
TT (nmol/L; to ng/dL: ×28.84)	0.89 ± 0.42^a^	0.35 ± 0.15^a^	<0.01
ADT (nmol/L; to ng/dL: ×29.04)	0.53 ± 0.25^a^	0.31 ± 0.13^a^	0.01
DHT (nmol/L; to ng/dL: ×29.04)	0.12 ± 0.05^a^	0.07 ± 0.03^a^	<0.01
11-OHAD (nmol/L; to ng/dL: ×30.24)	2.08 ± 1.11^a^	1.72 (1.10, 1.97)^b^	0.48
11-KAD (nmol/L; to ng/dL: ×30.04)	0.14 ± 0.10^a^	0.10 ± 0.06^a^	0.22
11-KT (nmol/L; to ng/dL: ×30.24)	1.14 ± 0.56^a^	0.98 ± 0.41^a^	0.41
11-OHT (nmol/L; to ng/dL: ×30.44)	0.10 (0.04, 0.13)^b^	0.08 ± 0.03^a^	0.45
EpiA (nmol/L; to ng/dL: ×29.04)	0.04 ± 0.02^a^	0.07 ± 0.06^a^	0.07
EpiT (nmol/L; to ng/dL: ×28.84)	0.04 ± 0.02^a^	0.01 (0.00, 0.01)^b^	<0.01
3α-diol (nmol/L; to ng/dL: ×29.25)	0.05 ± 0.04^a^	0.06 ± 0.05^a^	0.62
Estrogens			
E1 (pmol/L; to pg/mL: ×0.27)	79.50 ± 49.20^a^	19.30 ± 11.50^a^	<0.01
E2 (pmol/L; to pg/mL: ×0.27)	167.00 ± 90.20^a^	13.30 (8.44, 26.40)^b^	<0.01
E3 (pmol/L; to pg/mL: ×0.29)	2.39 (0.80, 5.58)^b^	1.32 (0.59, 3.64)^b^	0.30

a Data are mean ± SD.

b Data are median (25th–75th percentile). Group comparisons were performed using independent samples t-test or Mann-Whitney U test accordingly. *P* < 0.05 was considered statistically significant. Conversion factors from SI units to conventional units (ng/dL for most steroids, μg/dL for DHEA S, pg/mL for estrogens) are provided in parentheses next to each steroid in the Parameter column. For example, for DHEA (nmol/L), multiply by 28.84 to obtain ng/dL. AMH, anti-Müllerian hormone; INB, inhibin B; SHBG, sex hormone-binding globulin; FAI, free androgen index.

### 3.2 Changes in steroid hormone profiles following combined GnRHa and letrozole in the Group A

**Figure 2** displays box plots of the serum steroid hormones that showed significant changes over time in the Group A (n=13). The full dataset for this group is provided in **Supplementary Table 1**. In the classical androgen pathway, AD, TT, ADT, DHT, and EpiT decreased substantially from baseline to 1 month. They remained at lower levels at 6 months (all overall P < 0.01). Among 11‐oxygenated androgens, only 11‐KT exhibited a modest reduction at 1 month (overall *P* = 0.02), with no further change thereafter, whereas 11‐OHAD, 11‐KAD, and 11‐OHT showed no significant changes over time (all overall *P* > 0.05). In the backdoor pathway, 5α‐DHP, 17OH‐DHP, and 3α‐diol decreased significantly after treatment (all overall *P* < 0.01), whereas 17OH‐Allo did not change substantially (overall *P* = 0.38). FAI, a marker of bioavailable androgens, also decreased progressively over the 6‐month period (overall *P* < 0.01).

**Figure 2:**
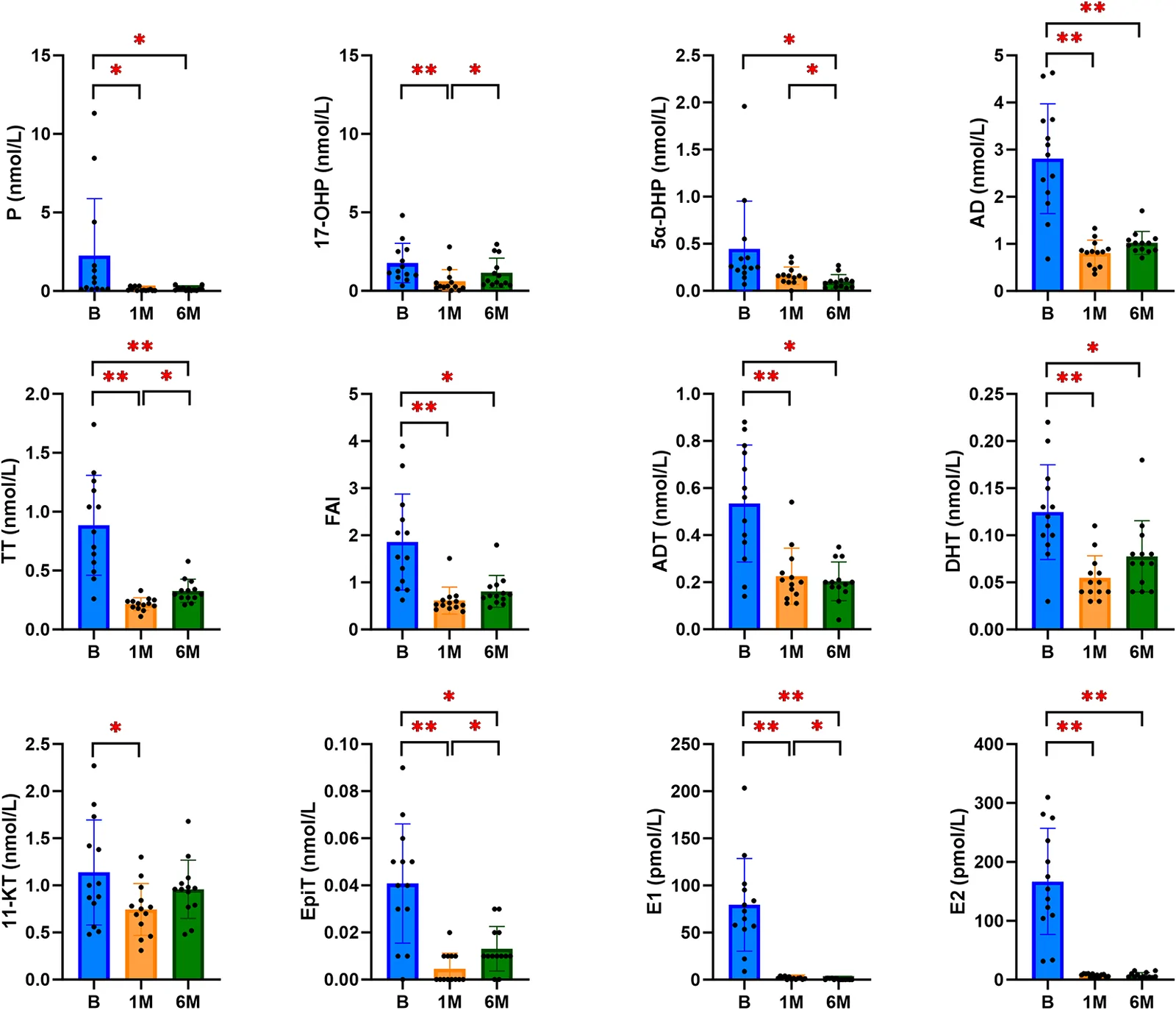
Box plots of serum steroid hormones with significant longitudinal changes in the Group A (n=13). Box plots showing the distribution of serum steroid hormones that changed significantly over time in girls receiving combined GnRHa and letrozole. Parameters include P, 17‐OHP, AD, TT, ADT, DHT, EpiT, 11‐KT, 5α‐DHP, E1, E2, and FAI. Data are presented as median (center line), interquartile range (box boundaries), and range (whiskers). Measurements were obtained at baseline, 1 month, and 6 months. Overall comparisons used repeated‐measures ANOVA or Friedman test; pairwise comparisons used paired t‐test or Wilcoxon test with Bonferroni correction. *P < 0.05, **P < 0.01. Full data are provided in **Supplementary Table 1**.

Among progestogens, serum P and 17‐OHP declined by 1 month and remained lower through 6 months (both overall *P* < 0.01), whereas PREG and 17‐OHPREG showed no significant changes (both overall *P* > 0.05). Serum E1 and E2 decreased markedly after treatment initiation (both overall *P* < 0.01) and remained at low levels throughout follow‐up, while E3 did not change significantly (overall *P* = 0.84). No significant overall time effects were observed for DHEA, DHEA‐S, or EpiA (all overall *P* > 0.05). Among reproductive hormones, AMH and INB decreased progressively over 6 months (both overall *P* < 0.01), whereas SHBG declined at 1 month and partially rebounded toward baseline by 6 months (overall *P* = 0.04).

In Group A, no patient had clinical hirsutism (modified Ferriman‐Gallwey score ≥6) or moderate‐to‐severe acne at baseline or at 6 months. Bone age increased from 12.53 ± 0.52 years at baseline to 12.83 ± 0.52 years at 6 months (mean change 0.30 years; paired t‐test, *P* < 0.01), which was less than the expected chronological age progression of 0.5 years over the same period.

### 3.3 Changes in steroid hormone profiles following add‐on letrozole in the Group B

**Supplementary Table 2** summarizes the longitudinal changes in all measured parameters over 6 months in the Group B (n=15), who received add‐on letrozole after established GnRHa monotherapy. Only a limited number of parameters showed significant overall changes. Among progestogens, PREG increased from baseline to 6 months (overall *P* = 0.02), and 17OH‐DHP rose at 6 months compared with both earlier time points (overall *P* < 0.01). In the androgen axis, only 3α‐diol showed a modest decrease at 1 month (overall *P* = 0.03). Serum E1 and E2 decreased significantly after letrozole add‐on (both overall *P* < 0.01). Among reproductive hormones, AMH declined progressively over 6 months (overall *P* < 0.01), while INB showed a modest decrease at 1 month (overall *P* = 0.02). No significant overall time effects were observed for the remaining parameters (all overall *P* > 0.05).

In Group B, no patient had clinical hirsutism (modified Ferriman‐Gallwey score ≥6) or moderate‐to‐severe acne at baseline or at 6 months. Bone age increased from 12.42 ± 0.93 years at baseline to 12.65 ± 0.91 years at 6 months (mean change 0.23 years; Wilcoxon signed‐rank test, *P* < 0.01), which was also less than the expected chronological age progression of 0.5 years over the same period.

## 4 Discussion

To our knowledge, this is the first study to systematically characterise the responses of classical, 11‐oxygenated, and backdoor androgen pathways to combined GnRHa and letrozole therapy in girls with advanced bone age. The comprehensive steroidomic approach employed here offers a broader endocrine safety assessment than previous reports based on isolated hormone measurements.

In Group A, classical androgens decreased substantially from baseline to 1 month, with effects maintained throughout the 6‐month observation period. Estrogens declined sharply and remained at low levels at 6 months. Progestogens P and 17‐OHP also decreased significantly, consistent with effective dual blockade of the HPG axis and peripheral aromatase. Notably, 11‐oxygenated androgens remained largely unchanged, with the exception of a modest and transient reduction in 11‐KT at 1 month. The stability of adrenal‐derived androgens suggests that adrenal function was not overtly affected. In the backdoor pathway, 5α‐DHP, 17OH‐DHP, and 3α‐diol decreased significantly, whereas 17OH‐Allo did not change substantially, suggesting only partial responsiveness to gonadal suppression. FAI also decreased progressively, consistent with a net reduction in bioactive androgens. In Group B, where HPG axis suppression had already been established, the addition of letrozole yielded limited changes. Most androgens and progestogens remained stable. The modest increases in PREG and 17OH‐DHP, without corresponding changes in downstream androgens, might reflect accumulation of upstream precursors. Estrogens decreased significantly in both groups, consistent with sustained suppression of estrogen biosynthesis regardless of baseline HPG axis activity. These hormonal changes were accompanied by a slower bone age advancement than expected. Regarding skeletal maturation, bone age advancement appeared slower than chronological age progression in both groups, with a mean increase of 0.30 years in Group A and 0.23 years in Group B, compared with the expected 0.5-year chronological progression. This may suggest that combined therapy has the potential to delay epiphyseal closure through dual suppression of estrogen signaling. Confirmation of its clinical relevance will require longer-term follow-up.

The efficacy of letrozole in suppressing estrogen-driven skeletal maturation has been demonstrated in girls with McCune-Albright syndrome, with significant reductions in bone age advancement and improvements in predicted adult height over long-term follow-up (17, 18). Recent retrospective data in girls with short stature and advanced bone age receiving rhGH, GnRHa, and letrozole showed improvements in predicted adult height without significant side effects, supporting the safety of letrozole-containing combination therapies (19). Mauras et al. (20) reported that letrozole monotherapy in boys led to elevated gonadotropins and supraphysiological testosterone increases. In the present study, testosterone decreased significantly throughout the 6‐month period, confirming that GnRHa effectively counteracts this rebound. The GAIL study (11, 12) demonstrated that combined leuprorelin and anastrozole in girls improved predicted adult height without hyperandrogenism or bone mineral abnormalities, though comprehensive steroid profiling was not performed. Our data extend these findings by delineating the responses of the backdoor and 11‐oxygenated pathways.

After GnRHa treatment, ovarian function is suppressed and estrogen levels are extremely low in girls, a physiological state that is partially comparable to that of postmenopausal women. Studies in postmenopausal women receiving letrozole have shown that letrozole reduces estrone and estradiol by 75–95%, with only a seemingly minor effect on adrenal steroidogenesis (21, 22). Among aromatase inhibitor users, levels of dihydrotestosterone, total testosterone, androstenedione, dehydroepiandrosterone, and the free androgen index did not differ significantly from those in healthy controls or untreated breast cancer patients (23). Notably, androgen-related side effects have been reported only with the steroidal aromatase inhibitor exemestane, whereas no such effects have been reported with the non-steroidal agents letrozole or anastrozole (24).

In the present study, AMH and INB decreased significantly in both groups after treatment. Previous studies have shown that AMH and INB levels decrease significantly in girls with CPP after 1 year of GnRHa treatment, and INB has been proposed as a reliable marker for monitoring treatment response (25). Hagen et al. found that AMH decreased significantly after 3 months of GnRHa treatment and returned to pretreatment levels after discontinuation, suggesting that this suppressive effect is reversible (26). These findings indicate that the decreases in AMH and INB reflect effective GnRHa-mediated suppression of ovarian follicular activity. The changes in SHBG were more complex. In Group A, SHBG decreased at 1 month and partially recovered by 6 months. SHBG is regulated by multiple factors, including sex hormones, insulin, and body composition. Previous studies have shown that SHBG levels are significantly lower in girls with CPP than in age-matched controls and decrease further during GnRHa treatment; however, these studies also noted that SHBG levels are mainly associated with body composition and DHEA-S rather than estrogen alone (27). Another study found that physiological estrogen concentrations do not participate in SHBG regulation in girls with CPP (28). Therefore, the transient decrease and partial recovery of SHBG observed in this study may reflect adaptive changes in metabolic status or body composition during treatment, rather than a direct consequence of estrogen suppression.

GnRHa adjunctive therapy has an overall favorable safety profile. Common adverse events include injection site pain, headache, hot flashes, and mood instability, which are usually mild and well tolerated. One real-world study reported that 61.1% of triptorelin-related adverse reactions occurred within 2 hours of administration, with severe events predominantly consisting of allergic reactions, hip synovitis, and arthralgia (29). However, in the context of combination therapy, the potential additive risk of GnRHa and letrozole co-suppressing estrogen warrants attention. Previous studies have shown that bone mineral density may decline modestly during GnRHa treatment, but bone mass accrual recovers after discontinuation, with peak bone mass appearing unaffected (30, 31). In the present study, no letrozole-related adverse events such as liver dysfunction, rash, or gastrointestinal symptoms were observed during the 6-month combination treatment, and no clinical or biochemical hyperandrogenism was observed. Given the dual suppression of estrogen, however, long-term monitoring of bone mineral density and growth parameters is warranted.

## 5 Limitations

Several limitations should be acknowledged. The sample size was relatively small, with 13 girls in Group A and 15 in Group B, which limits the generalizability of the findings. Group allocation was non-randomized and resulted in baseline differences between groups, which may have influenced the observed treatment effects. No adjustment for baseline differences was performed due to the small sample size. The follow-up duration was confined to 6 months, precluding assessment of long-term effects on bone age progression and final adult height, and it remains uncertain whether the observed slowing of bone age advancement is sustained beyond this period. Gonadotropin levels were not measured in parallel, limiting insight into HPG axis dynamics underlying the observed hormonal changes. The single-center design and the absence of a control group further restrict attribution of the observed effects to the intervention. Additionally, although LC-MS/MS provides high analytical specificity, inter-laboratory variability should be considered when comparing absolute values across studies.

## 6 Conclusions

This study employed high-sensitivity LC-MS/MS to evaluate dynamic changes in serum steroid profiles in girls with advanced bone age receiving combined GnRHa and letrozole therapy. In Group A, combined therapy significantly suppressed gonadal-derived androgens and estrogens, consistent with effective dual blockade, while adrenal-derived androgens remained stable. In Group B, where HPG axis suppression had already been established, the addition of letrozole yielded limited changes. Estrogens decreased significantly in both groups, and no clinical or biochemical evidence of hyperandrogenism was observed. Regarding clinical benefit, bone age advancement was slower than chronological age progression in both groups, suggesting that the combination may slow skeletal maturation; however, whether this translates into improved final adult height requires longer-term follow-up. These findings provide preliminary evidence for the short-term safety of this combination and offer endocrine-metabolic evidence to inform clinical decision-making, though longer-term follow-up in larger cohorts is needed.

## Data Availability

The raw data supporting the conclusions of this article will be made available by the authors, without undue reservation.
