# Supplementary material for: Effects of combined GnRHa and letrozole on serum steroid profiles in girls with advanced bone age
Source: Front Endocrinol (Lausanne). 2026 Oct 15;17:1984633. doi: 10.3389/fendo.2026.1984633 (PMC13716571; doi:10.3389/fendo.2026.1984633)
Supplement: Supplementary file 1 [file Table1.docx]

# Supplementary Table 1. Baseline and longitudinal changes in serum steroid hormones and bone age in Group A (n=13)

| **Parameter** | **Baseline** | **1 month** | **6 months** | ***P* value** | **B vs 1M** | **B vs 6M** | **1M vs 6M** |
| --- | --- | --- | --- | --- | --- | --- | --- |
| **Progestogens** |  |  |  |  |  |  |  |
| PREG (nmol/L; to ng/dL: ×31.65) | 0.68 ± 0.45 | 0.71 (0.34, 0.79) | 0.61 ± 0.41 | 0.79 | — | — | — |
| P (nmol/L; to ng/dL: ×31.45) | 0.54 (0.13, 3.02) | 0.06 (0.03, 0.25) | 0.10 (0.06, 0.19) | **<0.01** | **0.04** | **0.02** | 1.00 |
| 17-OHPREG (nmol/L; to ng/dL: ×33.25) | 2.20 (1.44, 4.87) | 3.52 ± 3.16 | 1.65 (0.99, 5.02) | 0.59 | — | — | — |
| 17-OHP (nmol/L; to ng/dL: ×33.05) | 1.79 ± 1.24 | 0.30 (0.18, 0.70) | 0.64 (0.39, 2.00) | **<0.01** | **<0.01** | 0.23 | **0.04** |
| 5α-DHP (nmol/L; to ng/dL: ×31.65) | 0.25 (0.21, 0.45) | 0.16 ± 0.09 | 0.09 (0.04, 0.11) | **<0.01** | 0.08 | **0.01** | **0.02** |
| 17OH-DHP (nmol/L; to ng/dL: ×31.25) | 0.00 (0.00, 0.03) | 0.00 (0.00, 0.00) | 0.02 ± 0.01 | **<0.01** | 0.08 | 0.52 | **<0.01** |
| 17OH-Allo (nmol/L; to ng/dL: ×33.43) | 0.12 ± 0.10 | 0.08 ± 0.05 | 0.10 ± 0.04 | 0.38 | — | — | — |
| **Androgens** |  |  |  |  |  |  |  |
| DHEA (nmol/L; to ng/dL: ×28.84) | 6.10 ± 3.54 | 5.86 ± 3.84 | 3.43 (2.43, 7.66) | 0.74 | — | — | — |
| DHEA-S (μmol/L; to μg/dL: ×36.85) | 1.99 (0.68, 2.51) | 1.73 (0.96, 1.97) | 2.06 ± 0.97 | 0.15 | — | — | — |
| AD (nmol/L; to ng/dL: ×28.64) | 2.81 ± 1.16 | 0.83 ± 0.28 | 1.02 (0.86, 1.09) | **<0.01** | **<0.01** | **<0.01** | 0.08 |
| TT (nmol/L; to ng/dL: ×28.84) | 0.89 ± 0.42 | 0.22 ± 0.05 | 0.33 ± 0.10 | **<0.01** | **<0.01** | **<0.01** | **0.02** |
| ADT (nmol/L; to ng/dL: ×29.04) | 0.53 ± 0.25 | 0.20 (0.14, 0.27) | 0.20 ± 0.08 | **<0.01** | **<0.01** | **0.02** | 1.00 |
| DHT (nmol/L; to ng/dL: ×29.04) | 0.12 ± 0.05 | 0.05 ± 0.02 | 0.07 (0.05, 0.09) | **<0.01** | **<0.01** | **0.02** | 0.10 |
| 11-OHAD (nmol/L; to ng/dL: ×30.24) | 2.08 ± 1.11 | 1.56 ± 0.81 | 1.99 ± 0.74 | 0.10 | — | — | — |
| 11-KAD (nmol/L; to ng/dL: ×30.04) | 0.14 ± 0.10 | 0.17 ± 0.08 | 0.15 (0.12, 0.21) | 0.40 | — | — | — |
| 11-KT (nmol/L; to ng/dL: ×30.24) | 1.14 ± 0.56 | 0.75 ± 0.28 | 0.96 ± 0.31 | **0.02** | **0.04** | 0.98 | 0.26 |
| 11-OHT (nmol/L; to ng/dL: ×30.44) | 0.10 (0.04, 0.13) | 0.05 (0.02, 0.09) | 0.08 ± 0.04 | 0.29 | — | — | — |
| EpiA (nmol/L; to ng/dL: ×29.04) | 0.04 ± 0.02 | 0.05 (0.02, 0.10) | 0.10 ± 0.07 | 0.58 | — | — | — |
| EpiT (nmol/L; to ng/dL: ×28.84) | 0.04 ± 0.02 | 0.00 (0.00, 0.01) | 0.01 ± 0.01 | **<0.01** | **<0.01** | **0.03** | **0.04** |
| 3α-diol (nmol/L; to ng/dL: ×29.25) | 0.05 ± 0.04 | 0.03 ± 0.02 | 0.04 ± 0.02 | 0.27 | — | — | — |
| **Estrogens** |  |  |  |  |  |  |  |
| E1 (pmol/L; to pg/mL: ×0.27) | 79.50 ± 49.20 | 1.73 ± 1.38 | 0.00 (0.00, 1.41) | **<0.01** | **<0.01** | **<0.01** | **0.02** |
| E2 (pmol/L; to pg/mL: ×0.27) | 167.00 ± 90.20 | 7.71 ± 2.46 | 5.66 (4.22, 11.57) | **<0.01** | **<0.01** | **<0.01** | 1.00 |
| E3 (pmol/L; to pg/mL: ×0.29) | 2.39 (0.80, 5.58) | 2.29 ± 2.15 | 2.60 ± 2.46 | 0.84 | — | — | — |
| **Reproductive hormones** |  |  |  |  |  |  |  |
| AMH（ng/mL） | 3.61 ± 1.62 | 1.59 ± 0.95 | 1.19 ± 0.78 | **<0.01** | **<0.01** | **<0.01** | 0.18 |
| INB（pg/mL） | 116.08 (65.68, 222.98) | 48.07 (40.19, 58.06) | 32.34 (23.95, 35.61) | **<0.01** | **<0.01** | **<0.01** | **<0.01** |
| SHBG（nmol/L） | 51.70 ± 18.71 | 37.60 ± 10.06 | 42.54 ± 10.10 | **0.04** | **0.03** | 0.14 | 0.47 |
| FAI | 1.86 ± 1.02 | 0.53 (0.46, 0.66) | 0.72 (0.61, 0.93) | **<0.01** | **<0.01** | **0.01** | 0.14 |
| **Bone age (years)** | 12.53 ± 0.52 | — | 12.83 ± 0.52 | **<0.01** | — | — | — |

Normally distributed data are presented as mean ± SD, whereas non-normally distributed data are presented as median (25th percentile, 75th percentile). For variables that were normally distributed at all three time points, repeated-measures ANOVA was used for overall comparison. When Mauchly's test of sphericity was significant (P < 0.05), the Greenhouse-Geisser correction was applied. Post-hoc pairwise comparisons were performed using the paired t-test with Bonferroni correction. For variables showing a non-normal distribution at any time point, the Friedman test was used for overall comparison, followed by post-hoc pairwise comparisons using the Wilcoxon signed-rank test with Bonferroni correction. All pairwise *P*-values reported were Bonferroni-corrected. For bone age, which was assessed at baseline and 6 months only, paired t-test was used for within-group comparison. *P*-values lower than 0.05 are marked in bold. "—" indicates not applicable. B, baseline; 1M, 1 month; 6M, 6 months.

# Supplementary Table 2. Baseline and longitudinal changes in serum steroid hormones and bone age in Group B (n=15)

| **Parameter** | **Baseline** | **1 month** | **6 months** | ***P* value** | **B vs 1M** | **B vs 6M** | **1M vs 6M** |
| --- | --- | --- | --- | --- | --- | --- | --- |
| **Progestogens** |  |  |  |  |  |  |  |
| PREG (nmol/L; to ng/dL: ×31.65) | 0.36 (0.24, 0.51) | **0.74 (0.38, 2.12)** | **0.29 ± 0.10** | **0.02** | 0.18 | 0.08 | **0.02** |
| P (nmol/L; to ng/dL: ×31.45) | 0.10 (0.03, 0.10) | 0.10 (0.06, 0.13) | 0.10 (0.06, 0.13) | 0.31 | — | — | — |
| 17-OHPREG (nmol/L; to ng/dL: ×33.25) | 1.41 (0.87, 3.19) | 1.33 (0.52, 1.95) | 0.64 (0.57, 0.75) | 0.27 | — | — | — |
| 17-OHP (nmol/L; to ng/dL: ×33.05) | 0.45 (0.39, 0.61) | 0.58 (0.22, 0.98) | 0.36 (0.21, 0.51) | 0.21 | — | — | — |
| 5α-DHP (nmol/L; to ng/dL: ×31.65) | 0.08 (0.06, 0.17) | 0.09 (0.04, 0.13) | 0.08 ± 0.05 | 0.45 | — | — | — |
| 17OH-DHP (nmol/L; to ng/dL: ×31.25) | 0.00 (0.00, 0.00) | 0.00 (0.00, 0.00) | 0.02 (0.01, 0.02) | **<0.01** | 0.95 | **<0.01** | **<0.01** |
| 17OH-Allo (nmol/L; to ng/dL: ×33.43) | 0.06 (0.00, 0.12) | 0.05 (0.00, 0.08) | 0.07 ± 0.04 | 0.28 | — | — | — |
| **Androgens** |  |  |  |  |  |  |  |
| DHEA (nmol/L; to ng/dL: ×28.84) | 4.09 (3.50, 7.66) | 5.43 ± 2.80 | 4.33 ± 1.56 | 0.55 | — | — | — |
| DHEA-S (μmol/L; to μg/dL: ×36.85) | 1.83 (1.53, 2.42) | 2.10 (1.66, 2.28) | 2.49 (2.41, 2.94) | 0.13 | — | — | — |
| AD (nmol/L; to ng/dL: ×28.64) | 1.14 (0.87, 1.31) | 0.98 (0.72, 1.10) | 1.03 ± 0.30 | 0.45 | — | — | — |
| TT (nmol/L; to ng/dL: ×28.84) | 0.35 ± 0.15 | 0.29 ± 0.10 | 0.34 ± 0.11 | 0.06 | — | — | — |
| ADT (nmol/L; to ng/dL: ×29.04) | 0.31 ± 0.13 | 0.33 (0.25, 0.35) | 0.34 (0.28, 0.36) | 0.59 | — | — | — |
| DHT (nmol/L; to ng/dL: ×29.04) | 0.07 ± 0.03 | 0.06 ± 0.02 | 0.07 ± 0.03 | 0.24 | — | — | — |
| 11-OHAD (nmol/L; to ng/dL: ×30.24) | 1.72 (1.10, 1.97) | 1.49 ± 0.54 | 1.32 (1.08, 1.59) | 0.25 | — | — | — |
| 11-KAD (nmol/L; to ng/dL: ×30.04) | 0.10 ± 0.06 | 0.13 ± 0.05 | 0.12 (0.08, 0.14) | 0.34 | — | — | — |
| 11-KT (nmol/L; to ng/dL: ×30.24) | 0.98 ± 0.41 | 0.87 ± 0.26 | 0.82 ± 0.34 | 0.46 | — | — | — |
| 11-OHT (nmol/L; to ng/dL: ×30.44) | 0.08 ± 0.03 | 0.06 ± 0.02 | 0.05 (0.04, 0.07) | 0.13 | — | — | — |
| EpiA (nmol/L; to ng/dL: ×29.04) | 0.07 ± 0.06 | 0.09 (0.01, 0.13) | 0.12 (0.05, 0.16) | 1.00 | — | — | — |
| EpiT (nmol/L; to ng/dL: ×28.84) | 0.01 (0.00, 0.01) | 0.01 (0.00, 0.01) | 0.01 (0.00, 0.01) | 0.80 | — | — | — |
| 3α-diol (nmol/L; to ng/dL: ×29.25) | 0.06 ± 0.05 | 0.03 ± 0.02 | 0.04 ± 0.02 | **0.03** | **0.03** | 0.30 | 0.46 |
| **Estrogens** |  |  |  |  |  |  |  |
| E1 (pmol/L; to pg/mL: ×0.27) | 19.30 ± 11.50 | 0.74 (0.00, 1.48) | 0.55 (0.00, 1.11) | **<0.01** | **<0.01** | **<0.01** | 1.00 |
| E2 (pmol/L; to pg/mL: ×0.27) | 13.30 (8.44, 26.40) | **7.60 (5.87, 9.58)** | **9.18 (7.38, 18.36)** | **<0.01** | 0.06 | 0.09 | 0.23 |
| E3 (pmol/L; to pg/mL: ×0.29) | 1.32 (0.59, 3.64) | 3.02 (0.62, 6.49) | 2.92 (0.83, 4.82) | 0.89 | — | — | — |
| **Reproductive hormones** |  |  |  |  |  |  |  |
| AMH（ng/mL） | 4.00 ± 2.85 | 1.75 ± 1.05 | 2.21 ± 2.34 | **<0.01** | **<0.01** | **0.04** | 1.00 |
| INB（pg/mL） | 62.64 (50.50, 114.11) | 53.24 (40.48, 63.10) | 56.14 (32.58, 61.12) | **0.02** | **0.02** | 0.08 | 1.00 |
| SHBG（nmol/L） | 28.82 (21.43, 40.67) | 29.32 (23.06, 36.28) | 27.35 (22.79, 40.22) | 0.34 | — | — | — |
| FAI | 0.97 (0.65, 1.46) | 0.91 (0.70, 1.38) | 1.15 (0.82, 1.32) | 0.42 | — | — | — |
| **Bone age (years)** | 12.42 ± 0.93 | — | 12.65 ± 0.91 | **<0.01** | — | — | — |

Normally distributed data are presented as mean ± SD, whereas non-normally distributed data are presented as median (25th percentile, 75th percentile). For variables that were normally distributed at all three time points, repeated-measures ANOVA was used for overall comparison. When Mauchly's test of sphericity was significant (P < 0.05), the Greenhouse-Geisser correction was applied. Post-hoc pairwise comparisons were performed using the paired t-test with Bonferroni correction. For variables showing a non-normal distribution at any time point, the Friedman test was used for overall comparison, followed by post-hoc pairwise comparisons using the Wilcoxon signed-rank test with Bonferroni correction. All pairwise P values reported were Bonferroni-corrected. For bone age, which was assessed at baseline and 6 months only, Wilcoxon signed-rank test was used for within-group comparison. P-values lower than 0.05 are marked in bold. "—" indicates not applicable. B, baseline; 1M, 1 month; 6M, 6 months.

# Supplementary Table 3. Method performance characteristics of steroid hormones.

| Analyte | Intra-assay CV(%) |  |  | Inter-assay CV (%) |  |  | Recovery(%) |  |  | Linear range | Unit | LOQ | LOD |
| --- | --- | --- | --- | --- | --- | --- | --- | --- | --- | --- | --- | --- | --- |
|  | Low | Med | High | Low | Med | High | Low | Med | High |  |  |  |  |
| PREG | 10.6 | 5.2 | 4.3 | 10.3 | 10.0 | 12.2 | 96 | 98 | 91 | 125-50000 | pg/mL | 125 | 31 |
| P | 9.9 | 3.7 | 3.5 | 7.3 | 7.0 | 9.8 | 94 | 96 | 93 | 0.125-50 | ng/mL | 0.0125 | 0.00625 |
| 11-DOC | 3.5 | 1.5 | 3.1 | 3.2 | 3.9 | 3.3 | 100 | 99 | 95 | 25-10000 | pg/mL | 2.5 | 1.25 |
| B | 6.2 | 2.1 | 3.7 | 7.3 | 8.1 | 9.5 | 97 | 95 | 97 | 0.05-20 | ng/mL | 0.005 | 0.0025 |
| 18-OHB | 1.6 | 2.1 | 1.8 | 3.6 | 2.2 | 3.3 | 95 | 107 | 102 | 50-20000 | pg/mL | 25 | 12.5 |
| ALD | 10.8 | 4.2 | 4.1 | 11.5 | 4.8 | 3.0 | 93 | 97 | 103 | 12.5-5000 | pg/mL | 12.5 | 5 |
| 17-OHPREG | 4.7 | 2.2 | 2.9 | 3.4 | 3.3 | 3.4 | 97 | 98 | 97 | 0.1-40 | ng/mL | 0.1 | 0.025 |
| 17-OHP | 6.4 | 1.9 | 2.1 | 4.8 | 4.2 | 4.2 | 109 | 101 | 97 | 0.05-20 | ng/mL | 0.005 | 0.0025 |
| 11-DOF | 2.7 | 2.1 | 2.3 | 3.2 | 2.4 | 2.3 | 105 | 100 | 100 | 50-20000 | pg/mL | 25 | 12.5 |
| 21-DOF | 3.3 | 1.5 | 3.6 | 4.4 | 2.6 | 2.5 | 103 | 97 | 100 | 25-10000 | pg/mL | 25 | 12.5 |
| F | 2.1 | 1.5 | 3.6 | 4.3 | 3.0 | 4.6 | 104 | 100 | 101 | 0.75-300 | ng/mL | 0.12 | 0.075 |
| 18-OHF | 3.0 | 1.7 | 1.9 | 4.2 | 2.1 | 3.2 | 99 | 99 | 104 | 25-10000 | pg/mL | 6.25 | 2.5 |
| 18-OF | 4.5 | 1.2 | 1.9 | 6.3 | 3.7 | 3.7 | 96 | 93 | 102 | 10-2000 | pg/mL | 10 | 5 |
| E | 2.1 | 1.3 | 3.5 | 2.1 | 2.3 | 2.9 | 105 | 99 | 100 | 0.125-50 | ng/mL | 0.0125 | 0.00625 |
| DHEA | 7.6 | 3.4 | 4.0 | 10.1 | 5.1 | 6.5 | 96 | 88 | 95 | 0.125-50 | ng/mL | 0.125 | 0.03 |
| DHEA-S | 0.8 | 2.1 | 2.4 | 4.7 | 3.1 | 3.1 | 101 | 98 | 98 | 12.5-5000 | ng/mL | 2.5 | 1.25 |
| AD | 1.7 | 2.0 | 2.0 | 2.3 | 1.8 | 2.3 | 102 | 101 | 100 | 50-20000 | pg/mL | 7.5 | 5 |
| 11-OHAD | 2.6 | 1.2 | 2.8 | 3.2 | 3.6 | 3.1 | 102 | 101 | 104 | 50-20000 | pg/mL | 50 | 25 |
| 11-KAD | 5.0 | 5.1 | 3.2 | 6.7 | 10.6 | 5.1 | 91 | 89 | 101 | 12.5-2500 | pg/mL | 25 | 6.25 |
| E1 | 2.5 | 3.8 | 2.9 | 9.9 | 6.0 | 5.8 | 81 | 90 | 95 | 5-2000 | pg/mL | 5 | 1 |
| TT | 2.4 | 1.5 | 2.6 | 3.3 | 2.7 | 2.0 | 102 | 100 | 100 | 50-20000 | pg/mL | 7.5 | 5 |
| 11-OHT | 4.1 | 2.6 | 2.5 | 5.8 | 4.9 | 3.1 | 109 | 102 | 105 | 12.5-5000 | pg/mL | 6.25 | 3 |
| 11-KT | 2.1 | 1.3 | 3.6 | 3.8 | 3.4 | 2.6 | 111 | 104 | 107 | 25-10000 | pg/mL | 5 | 2.5 |
| E2 | 8.7 | 5.6 | 3.4 | 15.5 | 14.7 | 5.7 | 77 | 91 | 101 | 5-2000 | pg/mL | 5 | 1 |
| E3 | 16.3 | 6.7 | 3.0 | 15.5 | 6.6 | 4.5 | 89 | 93 | 106 | 5-2000 | pg/mL | 5 | 1 |
| DHT | 8.0 | 1.6 | 2.0 | 6.6 | 4.8 | 4.0 | 98 | 97 | 96 | 25-10000 | pg/mL | 25 | 6.25 |
| EpiT | 5.7 | 2.3 | 3.8 | 4.9 | 4.8 | 4.8 | 118 | 115 | 110 | 25-10000 | pg/mL | 5 | 2.5 |
| ADT | 13.4 | 8.8 | 4.6 | 14.3 | 9.4 | 9.6 | 96 | 92 | 85 | 100-10000 | pg/mL | 100 | 50 |
| EpiA | 4.2 | 7.4 | 3.5 | 10.3 | 7.6 | 5.1 | 91 | 103 | 99 | 250-10000 | pg/mL | 250 | 100 |

CV: coefficient of variation; LOD: limit of detection; LOQ: limit of quantification; Med: medium.

# Supplementary Table 4. Gradient elution program for LC-MS/MS.

| **Step** | **Time (min)** | **Flow rate (mL/min)** | **Aqueous phase (%)** | **Organic phase (%)** | **Curve** |
| --- | --- | --- | --- | --- | --- |
| 1 | Initial | 0.3 | 60 | 40 | Initial |
| 2 | 0.50 | 0.3 | 60 | 40 | 6 |
| 3 | 4.00 | 0.3 | 45 | 55 | 6 |
| 4 | 7.50 | 0.3 | 25 | 75 | 6 |
| 5 | 8.20 | 0.3 | 10 | 90 | 6 |
| 6 | 8.30 | 0.5 | 2 | 98 | 6 |
| 7 | 9.00 | 0.5 | 2 | 98 | 6 |
| 8 | 10.00 | 0.3 | 60 | 40 | 1 |

Aqueous phase: 0.5 mM ammonium fluoride in water; Organic phase: methanol.

# Supplementary Table 5. Mass spectrometric parameters for steroid hormones.

| **Analyte** | **Ionization mode** | **Quantifier transition (m/z)** | **Collision energy (eV)** | **Cone voltage (V)** | **Retention time (min)** |
| --- | --- | --- | --- | --- | --- |
| PREG | ESI+ | 299.10 > 281.06 | 14 | 30 | 7.44 |
| P | ESI+ | 315.30 > 109.02 | 10 | 50 | 7.09 |
| 11-DOC | ESI+ | 331.30 > 109.06 | 15 | 40 | 5.74 |
| B | ESI+ | 347.30 > 121.08 | 18 | 50 | 4.68 |
| 18-OHB | ESI+ | 363.2 > 269.1 | 16 | 15 | 3.35 |
| ALD | ESI- | 359.23 > 189.11 | 18 | 20 | 2.97 |
| 17-OHPREG | ESI- | 331.08 > 287.16 | 20 | 50 | 6.14 |
| 17-OHP | ESI+ | 331.30 > 97.04 | 15 | 40 | 6.18 |
| 11-DOF | ESI+ | 347.30 > 109.05 | 16 | 50 | 4.86 |
| 21-DOF | ESI+ | 347.27 > 311.23 | 14 | 50 | 4.45 |
| F | ESI+ | 363.30 > 327.19 | 7 | 50 | 3.77 |
| 18-OHF | ESI+ | 379.2 > 267.2 | 28 | 18 | 2.61 |
| 18-OF | ESI+ | 377.2 > 313.2 | 40 | 20 | 2.28 |
| E | ESI+ | 361.23 > 121.04 | 32 | 50 | 3.4 |
| DHEA | ESI+ | 271.23 > 213.20 | 15 | 40 | 6.01 |
| AD | ESI+ | 287.30 > 109.06 | 13 | 50 | 5.43 |
| 11-OHAD | ESI+ | 303.2 > 145.2 | 40 | 26 | 4.18 |
| 11-KAD | ESI+ | 301.2 > 121.1 | 30 | 20 | 3.57 |
| TT | ESI+ | 289.30 > 109.08 | 12 | 50 | 5.91 |
| 11-OHT | ESI+ | 305.2 > 269.2 | 14 | 14 | 4.58 |
| 11-KT | ESI+ | 303.2 > 121.1 | 40 | 22 | 3.98 |
| DHT | ESI+ | 291.30 > 255.28 | 16 | 50 | 6.66 |
| E1 | ESI- | 269.20 > 145.04 | 38 | 40 | 5.26 |
| E2 | ESI- | 271.04 > 183.06 | 40 | 30 | 5.33 |
| E3 | ESI- | 287.04 > 171.06 | 35 | 50 | 2.58 |
| EpiT | ESI+ | 289.3 > 97.1 | 40 | 20 | 6.79 |
| ADT | ESI+ | 273.3 > 147.2 | 30 | 20 | 7.54 |
| EpiA | ESI+ | 273.3 > 147.2 | 30 | 20 | 6.43 |
| DHEA-S | ESI- | 367.17 > 80.00 | 62 | 50 | 3.54 |

# Supplementary Table 6. Chemicals, reagents, standards and internal standards.

| **Category** | **No.** | **Name** | **Manufacturer** | **Catalog No.** |
| --- | --- | --- | --- | --- |
| **Reagents** | 1 | Methanol (HPLC grade, 4L) | Thermo Fisher | A452-4 |
|  | 2 | Acetonitrile (HPLC grade, 4L) | Thermo Fisher | A998-4 |
|  | 3 | Water (distilled) | Watsons | NA |
|  | 4 | Ammonium fluoride (25 g/bottle) | Sigma-Aldrich | 338869 |
| **Standards** | 1 | PREG | Cambridge | ULM-9159-0.01 |
|  | 2 | P | Sigma | V900699 |
|  | 3 | 11-DOC | IsoSciences | 13406 |
|  | 4 | B | TRC | C695700 |
|  | 5 | 18-OHB | Cerilliant | H-106 |
|  | 6 | ALD | Cerilliant | A-096 |
|  | 7 | 17-OHPREG | Cambridge | ULM-9155-D |
|  | 8 | 17-OHP | TRC | H952330 |
|  | 9 | 11-DOF | IsoSciences | 9012UNL |
|  | 10 | 21-DOF | Cambridge | ULM-9987-0.001 |
|  | 11 | F | TRC | TRC |
|  | 12 | 18-OHF | CMSS | CMS-T3100360 |
|  | 13 | 18-OF | TRC | O870615 |
|  | 14 | E | Dr Ehrenstorfer | C11705400 |
|  | 15 | DHEA | Cambridge | ULM-9143-0.01 |
|  | 16 | DHEA-S | Medical Isotopes | 30930 |
|  | 17 | AD | Dr Ehrenstorfer | C10255030 |
|  | 18 | 11-OHAD | Steraloids Inc | A6630 |
|  | 19 | 11-KAD | Steraloids Inc | A7250 |
|  | 20 | TT | Dr Ehrenstorfer | C17322500 |
|  | 21 | 11-OHT | Steraloids Inc | A5760 |
|  | 22 | 11-KT | Steraloids Inc | A6720 |
|  | 23 | DHT | Cerilliant | D-073 |
|  | 24 | E1 | TRC | E889050 |
|  | 25 | E2 | Dr Ehrenstorfer | C13213100 |
|  | 26 | E3 | Dr Ehrenstorfer | C13213200 |
|  | 27 | EpiT | A ChemTek Inc. | 1ST2266 |
|  | 28 | ADT | A ChemTek Inc. | 1ST8508 |
|  | 29 | EpiA | A ChemTek Inc. | 1ST2201-100A |
| **Internal standards** | 1 | PREG-D4 | Cambridge | DLM-6896-0.01 |
|  | 2 | P-D9 | Cerilliant | P-070 |
|  | 3 | 11-DOC-D8 | Medical Isotopes | D4301 |
|  | 4 | 18-OHB-D4 | IsoSciences | S8066-0.1 |
|  | 5 | ALD-D7 | IsoSciences | 5093 |
|  | 6 | 17-OHPREG-D2 C2 | Cambridge | CDLM-9154-0.001 |
|  | 7 | 17-OHP-D8 | CNW | D-5650 |
|  | 8 | 11-DOF-D5 | IsoSciences | 9012 |
|  | 9 | 21-DOF-D8 | Cambridge | DLM-8305-0.01 |
|  | 10 | F-D4 | TRC | C696302 |
|  | 11 | 18-OHF-D4 | Cambridge | DLM-10006-C |
|  | 12 | E-D8 | TRC | C696502 |
|  | 13 | DHEA-D6 | IsoSciences | 5170 |
|  | 14 | DHEA-S-D6 | Medical Isotopes | D17974 |
|  | 15 | AD-C3 | Sigma | 730645 |
|  | 16 | 11-OHAD-D4 | IsoSciences | 16148 |
|  | 17 | TT-C3 | Cerilliant | T-070 |
|  | 18 | 11-KT-D3 | IsoSciences | 16144 |
|  | 19 | DHT-C3 | Sigma | 730637 |
|  | 20 | E1-D4 | Medical Isotopes | D171 |
|  | 21 | E2-D4 | Cambridge | DLM-2487-5 |
|  | 22 | E3-D3 | Cambridge | DLM-8586-0.005 |
|  | 23 | ADT-D4 | CMASS | 361432-60-2 |

Dr. Ehrenstorfer (Dr. Ehrenstorfer GmbH, Germany); Cerilliant (Cerilliant Corporation, USA); TRC (Toronto Research Chemicals, Canada); IsoSciences (IsoSciences, USA); Cambridge (Cambridge Isotope Laboratories, USA); Medical Isotopes (Medical Isotopes, USA); Steraloids Inc (Steraloids Inc., USA); A ChemTek Inc. (A ChemTek Inc., USA); CMASS (CMASS Chemical Reagents, China); CNW (CNW Technologies, Germany); Sigma/Sigma-Aldrich (Merck KGaA, Germany); Thermo Fisher (Thermo Fisher Scientific, USA); Watsons (Watsons Water, China).
